# *Clostridium difficile* 027/BI/NAP1 Encodes a Hypertoxic and Antigenically Variable Form of TcdB

**DOI:** 10.1371/journal.ppat.1003523

**Published:** 2013-08-01

**Authors:** Jordi M. Lanis, Latisha D. Heinlen, Judith A. James, Jimmy D. Ballard

**Affiliations:** 1 Department of Microbiology and Immunology, The University of Oklahoma Health Sciences Center, Oklahoma City, Oklahoma, United States of America; 2 Department of Medicine, The University of Oklahoma Health Sciences Center, Oklahoma City, Oklahoma, United States of America; 3 Arthritis and Clinical Immunology Research Program, Oklahoma Medical Research Foundation, Oklahoma City, Oklahoma, United States of America; University of California Los Angeles, United States of America

## Abstract

The *Clostridium difficile* exotoxin, TcdB, which is a major virulence factor, varies between strains of this pathogen. Herein, we show that TcdB from the epidemic BI/NAP1/027 strain of *C. difficile* is more lethal, causes more extensive brain hemorrhage, and is antigenically variable from TcdB produced by previously studied strains of this pathogen (TcdB_003_). In mouse intoxication assays, TcdB from a ribotype 027 strain (TcdB_027_) was at least four fold more lethal than TcdB_003_. TcdB_027_ caused a previously undescribed brain hemorrhage in mice and this correlated with a heightened sensitivity of brain microvascular endothelial cells to the toxin. TcdB_003_ and TcdB_027_ also differed in their antigenic profiles and did not share cross-neutralizing epitopes in a major immunogenic region of the protein. Solid phase humoral mapping of epitopes in the carboxy-terminal domains (CTD) of TcdB_027_ and TcdB_003_ identified 11 reactive epitopes that varied between the two forms of TcdB, and 13 epitopes that were shared or overlapping. Despite the epitope differences and absence of neutralizing epitopes in the CTD of TcdB_027_, a toxoid form of this toxin primed a strong protective response. These findings indicate TcdB_027_ is a more potent toxin than TcdB_003_ as measured by lethality assays and pathology, moreover the sequence differences between the two forms of TcdB alter antigenic epitopes and reduce cross-neutralization by antibodies targeting the CTD.

## Author Summary

During the past decade, the *C. difficile* BI/NAP1/027 strain has emerged and in some settings predominated as the cause of *C. difficile* infection. Moreover, in some reports *C. difficile* BI/NAP1/027 has been associated with more severe disease. The reasons for association of this strain with more severe disease and relapse are poorly understood. We compared the toxicity and antigenic profiles of the major *C. difficile* virulence factor, TcdB, from a previously studied reference strain and a BI/NAP1/027 strain. The results indicate TcdB_027_, the toxin from the BI/NAP1/027 strain, is more lethal and causes more extensive brain hemorrhaging than TcdB_003_, the toxin produced by a reference strain of *C. difficile*. Furthermore, the results show that the antigenic carboxy-terminal domain (CTD) encodes at least 11 epitopes that differ between the two forms of TcdB. In line with this, experiments demonstrate that antiserum against the CTD does not cross-neutralize TcdB_003_ and TcdB_027_ toxicity against CHO cells, and TcdB_027_ appears to be devoid of neutralizing epitopes in this domain. These findings indicate differences in TcdB_003_ and TcdB_027_ contribute to increased virulence of *C. difficile* BI/NAP1/027 and reduce the likelihood of acquired immunity providing cross-protection against infection by these strains.

## Introduction

*Clostridium difficile* is the leading cause of hospital-acquired diarrhea in developed countries [1], [2], [3], [4]. This spore-forming anaerobic bacterium contaminates hospital environments and infects patients undergoing antibiotic therapy within health care facilities [2], [5], [6]. Despite these problems, historically, treatment with antibiotics such as metronidazole and vancomycin has been an effective means of treating this disease [7], [8]. Yet, disturbing trends of increased morbidity and mortality, as well relapse of *C. difficile* infected patients have become apparent over the past decade [9], [10], [11], [12], [13], [14], [15]. These trends correlate with the emergence of the BI/NAP1/027 strain of *C. difficile* [10], [12], [16], [17]. Although an absolute association between BI/NAP1/027 strains and increased disease severity has not been made in all cases [18], [19], [20], [21], extensive clinical surveillance over the past ten years has shown a strong correlation between BI/NAP1/027 frequency and mortality rate [22], [23]. This *C. difficile* strain has now been found in a majority of states in the US and is prominent both in Europe and Canada [16], [24]. To date, many factors such as antibiotic resistance, sporulation ability, and toxin production have been proposed to contribute to the potential difference in virulence of historical ribotypes and *C. difficile 027* [13], [25], [26], [27], [28], [29]. Yet, the relevance of these factors is still greatly debated [30], [31], leaving us with a poor understanding into how this emergent strain correlates with increased mortality.

*C. difficile* produces two large clostridial toxins, TcdA and TcdB, which cause extensive tissue damage and are major virulence factors in human disease [32], [33], [34]. Our work has focused on understanding how variations in the toxins produced by historical and epidemic strains change the extent of *C. difficile* virulence [35], [36]. Of particular interest are the differences in the sequence and activities of TcdB, which has been implicated as a critical *C. difficile* virulence factor [37], [38]. We hypothesize that variation between TcdB from previously predominant ribotypes and BI/NAP1/027 strains, is a major contributing factor to the increased virulence of the recently emerged forms of *C. difficile*.

TcdB (∼270 kDa; 2366 amino acids; YP_001087135.1) is a single chain polypeptide toxin where the glucosyltransferase domain is located at the N-terminus (GTD: 1–543), followed by an autoprocessing site between amino acid 543 and 544 which is subject to intramolecular cleavage by the cysteine protease domain (CPD: 544–807), a hydrophobic transmembrane domain (TMD: 956–1128), and a putative receptor binding domain at the C-terminus (CTD: 1651–2366) [39], [40], [41], [42], [43], [44], [45]. The gene encoding TcdB is located within a pathogenicity locus on the chromosome of *C. difficile* along with genes encoding TcdA (enterotoxin; YP_001087137.1), TcdE (YP_00108136.1), and regulators of toxin gene expression (TcdC, YP_001087138.1 and TcdR, YP_00108134.1) [46]. While the sequence of TcdA, TcdE, TcdR, and TcdC are almost identical between ribotype 012/003 and BI/NAP1/027 strains, TcdB is more variable (96% similarity, 92% identity) [35]. These differences in the sequence of TcdB may explain the observations of Wren and colleagues, who found that TcdB from a BI/NAP1/027 strain (TcdB_027_) is more potent on cultured cells than TcdB from a historical ribotype 012 strain [47]. In line with this we also found that TcdB_027_ causes more extensive and broader tissue pathologies than TcdB from the commonly referenced strain, VPI 10463 (TcdB_003_), in a zebrafish embryo model [35]. As a possible underlying mechanism for these differences in activity, we found previously that TcdB_027_ is translocated into cells more rapidly and is autoprocessed more efficiently than TcdB_003_ [35].

The greatest sequence variation between the two forms of TcdB is found in the C-terminal domain (CTD), which we define as the region of the toxin between amino acid 1651 and the terminal residue at position 2366. There is an overall 88% sequence identity between TcdB_027_1651-2366 and TcdB_003_1651-2366. The CTD of TcdB encodes combined repetitive oligopeptides (CROPs), which are thought to be responsible for the recognition of glycans on target cells [39], [48], and as such the CTD is often referred to as the receptor binding domain. However, the role of the CTD as the receptor binding domain is still very much debated as no receptor has been identified, and studies in TcdA have shown that this region contributes to, but is not required for cellular uptake of the toxin [49]. The CTD is also antigenic and known to contain neutralizing epitopes [50], [51]. Yet, whether sequence differences in the CTD of TcdB_027_ and TcdB_003_ alter the tropism or antigenic profiles of these two forms of the toxin is not known.

In the current study, we examined differences in the lethality and in vivo pathologies of TcdB_027_ and TcdB_003_. The data indicate TcdB_027_ exhibits a lethal dose substantially lower than TcdB_003_. We also show that while both toxins caused pronounced hemorrhaging in major organs, TcdB_027_ caused brain pathologies in vivo, as well as an increased cytotoxicity on brain microvascular cells in vitro. This study also characterized the influence of the CTD on this cell tropism and the possible contribution of sequence variation to changes in antigenicity. The data suggest that the CTD may not occupy the same role in TcdB_027_ as TcdB_003_, and identifying these key differences is a critical step toward understanding the virulence and systemic effects of *C. difficile* associated disease.

## Results

### TcdB_027_ Exhibits a Lower Lethal Dose Than TcdB_003_

**Figure 1:**
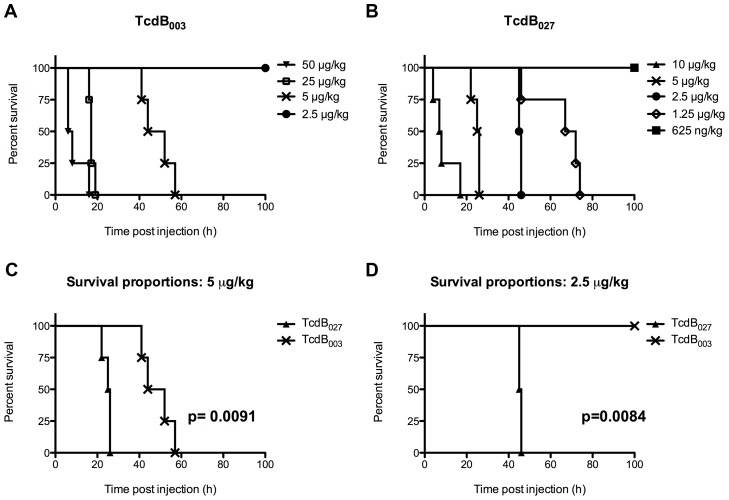
Comparative survival curves of mice injected with TcdB_003_ and TcdB_027._ Kaplan-Meier graphs showing the time to death of BALB/c mice that were injected intravenously with TcdB. (A) Survival time of mice (n = 4) injected with 50 ug/kg, 25 ug/kg, 5 ug/kg, and 2.5 ug/kg of TcdB_003_. (B) Survival time of mice (n = 4) injected with 10 ug/kg, 5 ug/kg, 2.5 ug/kg, 1.25 ug/kg, and 625 ng/kg of TcdB_027_. (C) Kaplan-Meier graph comparing the time to death of mice injected with 5 ug/kg of TcdB_003_ or TcdB_027_. The difference between the curves is indicated by the p value determined from a log-rank analysis. (D) Kaplan-Meier graph comparing the time to death of mice injected with 2.5 ug/kg of TcdB_003_ or TcdB_027_. The difference between the curves is indicated by the p value determined from a log-rank analysis.

In previous work we found that that TcdB_027_ is more cytototoxic and causes broader tissue damage in a zebrafish embryo model than TcdB_003_ [35]. To determine how this difference in activity might impact systemic damage and lethality between the two forms of the toxin, in the first set of experiments in this study we determined and compared the lethal doses of TcdB_003_ and TcdB_027_ in a murine systemic intoxication model. The previously published lethal dose of 220 µg/kg (i.p.) for TcdB_003_ [32] was used to establish a range of toxin concentrations for these treatments, but the lethality we observed via i.v. injection was much higher than previously reported. As a result, the initial doses of 100 µg/kg (data not shown), 50 µg/kg, and 25 µg/kg of TcdB_003_ were much more potent than anticipated, and resulted in a very rapid time to death (Fig. 1A). Therefore, the remaining mice were subjected to much lower doses of 5 µg/kg and 2.5 µg/kg of TcdB_003_. Based on the results of the TcdB_003_ treated mice, the TcdB_027_ group started with a dose of 10 µg/kg and was continued with 1∶2 dilutions down to 625 ng/kg of TcdB_027_. After the mice were injected with TcdB_003_ or TcdB_027_, they were followed for up to 7 days and the survival curves of the data from these experiments are shown in Fig. 1B.

The data shown in Fig. 1 indicate mice injected with TcdB_027_ succumb to the toxin at a lower dose than that observed in mice injected with TcdB_003_. Within 26 h of treatment all of the mice administered 5 µg/kg of TcdB_027_ died or reached a moribund condition. In comparison, mice administered the same dose of TcdB_003_ did not succumb to the toxin until after 40 h and as long as 57 h with a median survival of 48 hr (Fig. 1C). At the next lower dose (2.5 µg/kg), no mice survived TcdB_027_ treatment, while all of the mice treated with TcdB_003_ survived (Fig. 1D). Based on these outcomes we estimated the LD_50_ of TcdB_027_ to be between 625 ng/kg and 1.25 µg/kg of body weight. In comparison, a higher range for TcdB_003_ was estimated and fell between 2.5 µg/kg and 5 µg/kg of body weight. Thus, in line with previous studies demonstrating more potent effects on cultured cells and zebrafish embryos, TcdB_027_ also appears to be more toxic than TcdB_003_ in a rodent model of intoxication.

### TcdB_027_, but not TcdB_003_ Causes Extensive Brain Hemorrhaging

**Figure 2:**
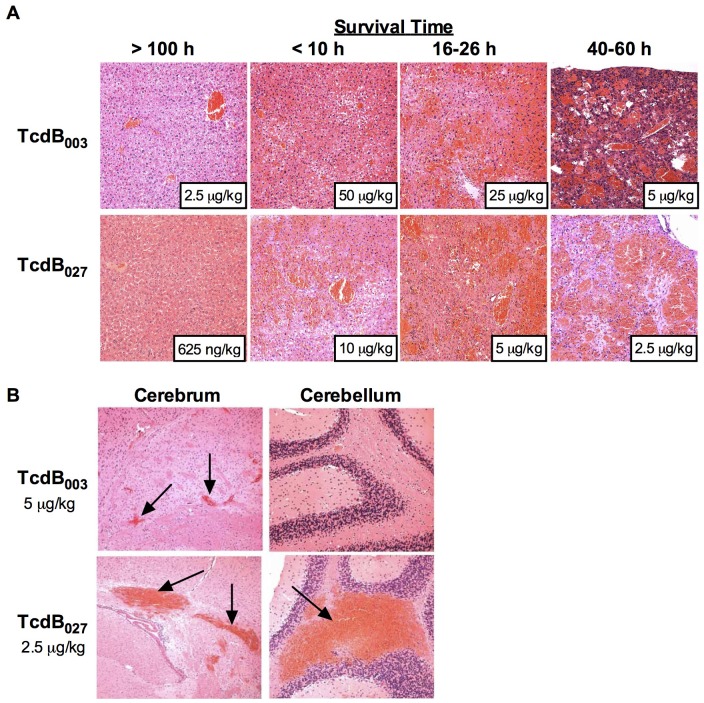
In vivo pathologies of TcdB_003_ and TcdB_027_. (A) Top- Liver pathologies from BALB/c mice injected with (from left to right) 2.5 µg/kg, 50 µg/kg, 25 µg/kg, and 5 µg/kg of TcdB_003_. Bottom- Liver pathologies from BALB/c mice injected with (from left to right) 625 ng/kg, 10 µg/kg, 5 µg/kg, or 2.5 µg/kg of TcdB_027_. All photos are a 20× magnification of H&E stained sections and are listed by survival time. (B) Pathologies of the cerebrum and cerebellum with arrows pointing to areas of hemorrhaging. Representative photos (20×) of H&E stained sections from BALB/c mice injected with 5 µg/kg TcdB_003_ (top) or 2.5 µg/kg of TcdB_027_ (bottom).

The results shown in Fig. 1, combined with our earlier findings in the zebrafish model [35], all point to the fact that TcdB_027_ is more toxic than TcdB_003_. Recent work by Steele and colleagues detected TcdA and TcdB circulating in the bloodstream of piglets infected by *C. difficile*, and this correlated with systemic effects that could be blocked by passive administration of antibodies against the toxins [52]. This led us to question whether TcdB_027_ might also cause more extensive systemic damage than TcdB_003_ due to its higher potency. To assess this, mice were administered TcdB_003_ (2.5 µg/kg to 50 µg/kg) or TcdB_027_ (625 ng/kg to 10 µg/kg) and tissue pathologies were examined. Tissues and organs from mice administered sublethal doses of the toxins did not reveal pathologies that differed from that of control (Fig. 2A). In contrast, abnormal tissue histologies were found in several of the major organs examined from mice intoxicated with lethal doses of TcdB. Mice treated with either TcdB_003_ or TcdB_027_ showed pronounced liver damage with extensive blood-pooling, parenchymal cell loss, and evidence of hemorrhage, which can be visualized by the appearance and expansion of the dark red patches as the survival time progresses (Fig. 2A). To a lesser extent, acute hepatocellular coagulative necrosis and hemorrhage in the spleen along with follicular necrosis and possible apoptotic cells was also detected (data not shown). The severity of the observed pathologies was more related to the length of time of toxin exposure rather than toxin concentration. Figure 2A shows representative liver sections from TcdB_003_ and TcdB_027_ treated mice, illustrating that the damage is the more extensive in mice receiving the minimum lethal dose and surviving for the longest period of time.

Despite the difference in lethality, the majority of the in vivo effects of TcdB_003_ and TcdB_027_ were identical, with the exception of moderate to severe hemorrhage detected in the brain of TcdB_027_ treated mice. Indeed, brain hemorrhage was the most obvious difference between mice exposed to the two forms of TcdB. The brains of mice treated with TcdB_003_ displayed only small lesions while the brain hemorrhage of TcdB_027_-treated mice was profuse with large multi-focal areas of blood accumulation within the cerebellum and cerebrum (Fig. 2B). These data suggest there may be a loss of endothelial integrity in mice challenged with TcdB, as well as a significant difference in the in vivo targeting and tropism of TcdB_003_ versus TcdB_027_.

### TcdB_027_ Is More Toxic Than TcdB_003_ to Brain Microvascular Endothelial Cells

**Figure 3:**
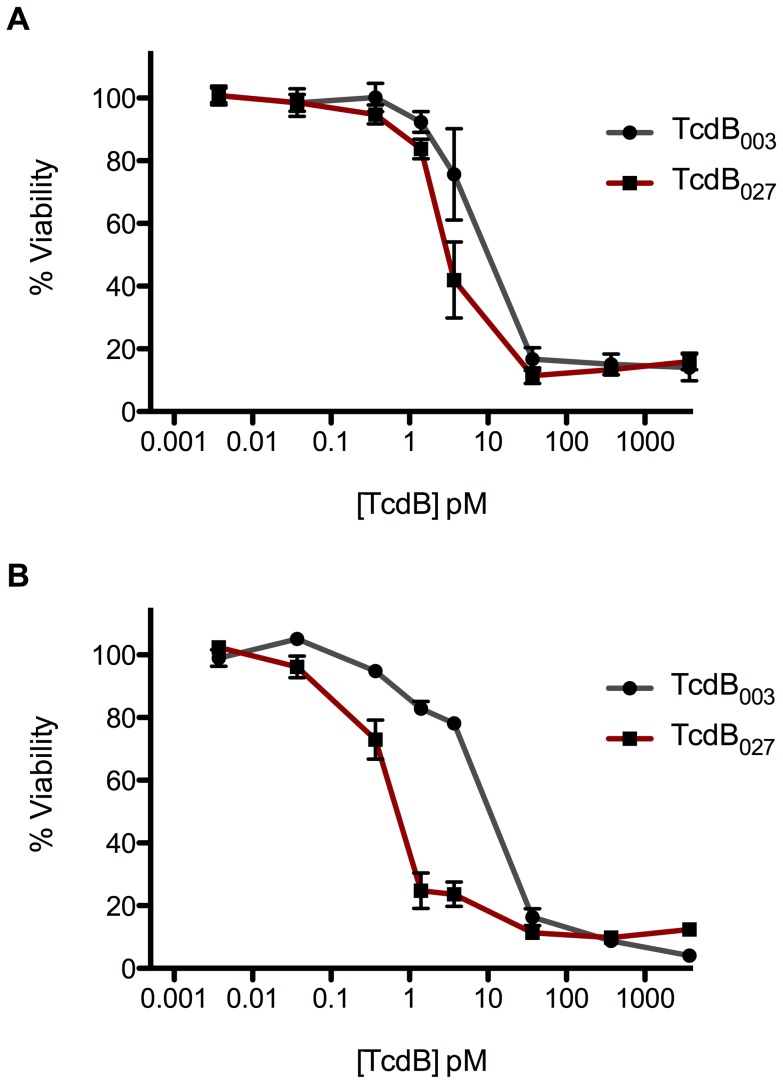
In vitro cytotoxicity of TcdB_003_ and TcdB_027_ on endothelial cells. Rat aortic endothilial cells (A) or rat brain microvascular endothelial cells (B) were exposed to 10-fold dilutions of TcdB_003_ (gray) or TcdB_027_ (red) from 3.7 nM to 3.7 fM for 24 h and cell viability was determined by WST-8 staining. The error bars represent the standard deviation from the mean of three biological replicates containing three technical replicates.

Experiments were next performed to determine the toxicity of the two forms of the TcdB on endothelial cell lines as a possible correlation with the differences in the amount of brain hemorrhage. We first wanted to determine whether endothelial cells displayed increased sensitivity to TcdB compared to the epithelial-like cells (e.g. CHO cells) that are normally used in cytotoxicity assays. Rat Aortic Endothelial Cells (RAEC) exposed to TcdB_003_ and TcdB_027_ displayed very similar cytotoxic doses (Fig. 3A). The concentration needed to cause toxicity in 50% of culture cells (TCD_50_) for TcdB_003_ was 6.07±1.41×10^−12^ M and 2.74±1.16×10^−12^ M for TcdB_027_. Since the major differences in pathology between TcdB_003_ and TcdB_027_ occurred in the brain, we next tested rat brain microvascular endothelial cells (RBMVEC) for differences in sensitivity to the two forms of TcdB. Interestingly, there was a 10-fold difference in the cytotoxicity of TcdB_027_ on the RBMVECs, with the TCD_50_ being 6.32±1.16×10^−13^ M compared to the TCD_50_ of 8.46±1.12×10^−12^ M for TcdB_003_ (Fig. 3B). These data indicated that TcdB was highly cytotoxic on endothelial cells, as the previous published observations of TcdB_003_ and TcdB_027_ toxicity on CHO cells is 2.53×10^−11^ and 2.37×10^−13^ respectively. Additionally, the RBMVECs had a greater susceptibility to TcdB_027_, which correlates with the brain pathologies in Fig. 2B.

### The Carboxy-Terminal Domains of TcdB_003_ and TcdB_027_ (CTD_003_ and CTD_027_) Differ in Cell Interactions and Their Susceptibility to Antibody Neutralizations

To further study the differences in the cell and organ targeting between TcdB_003_ and TcdB_027_, we focused on the CTD, which is thought to be important in facilitating cell interactions [39], [53]. We hypothesized that if this region is indeed important in cell targeting, then the sequence differences between TcdB_003_ and TcdB_027_ in this region could be an important factor in the distinct cell tropism and animal pathologies between the toxins. We also predicted that these differences could change the profile of antigenic epitopes, and perhaps neutralizing epitopes, in the CTD. We designed a set of experiments to address both of these possibilities.

In order to evaluate differences in the CTD of TcdB_003_ and TcdB_027_ we expressed and purified protein fragments representing this region of each toxin. These fragments consisted of the final 721 amino acids of the TcdB protein, including the CROP region along with approximately 206 residues amino terminal to the CROP region. Based on previous sequence comparisons, there are 89 residues that differ between CTD_003_ and CTD_027_ [35].

**Figure 4:**
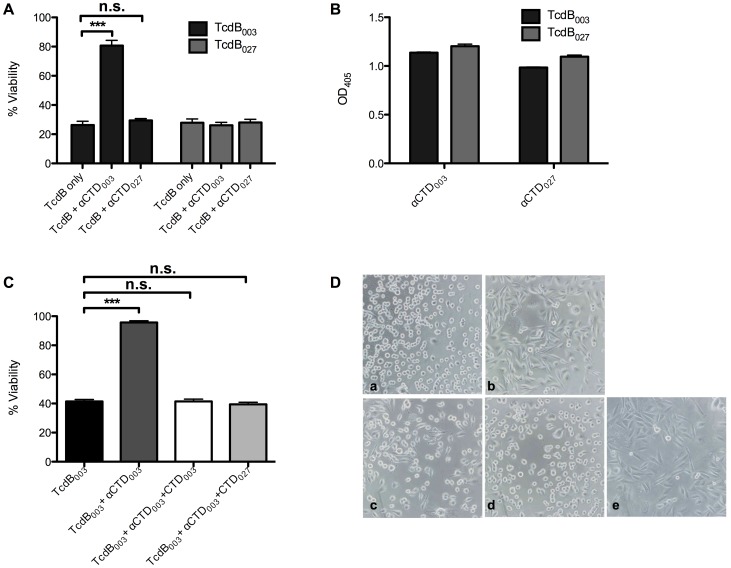
Neutralization of TcdB with αCTD antiserum. (A) Percent viability of CHO cells treated for 24 hrs with 37 pM of TcdB_003_ (black) or TcdB_027_ (gray) alone or after preincubation for 1 h with αCTD_003_ antiserum or αCTD_027_ antiserum. Cell viability was determined by WST-8 staining and the error bars represent the standard deviation from the mean of three biological replicates containing three technical replicates. *** p<0.001 (B) ELISA data showing the specificity of the αCTD antibodies to TcdB_003_ (black) and TcdB_027_ (gray) as measured by the optical density at 405 nm. The error bars represent the standard deviation from the mean of three samples. (C) Percent viability of CHO cells treated for 24 hrs with 37 pM TcdB_003_ alone (black) or combined with αCTD_003_ antiserum (dark gray), or with αCTD_003_ antiserum plus 3.7 nM of the CTD_003_ (white) or CTD_027_ (light gray) protein fragments. Cell viability was determined by WST-8 staining and the error bars represent the standard deviation of three biological replicates containing three technical replicates. *** p<0.001 (D) Representative phase contrast photographs of CHO cells after 6 h exposure to (a) 370 pM of TcdB_003_ alone or 370 pM TcdB_003_ with (b) 1∶100 CTD_003_ antiserum or 1∶100 CTD_003_ antiserum plus (c) excess CTD_003_ or (d) CTD_027_ (e) untreated control.

Initially, each CTD was used as an antigen to immunize rabbits for the collection of CTD antisera, which were then used in TcdB neutralization assays to further determine the impact of the CTD on the activity of both TcdB_003_ and TcdB_027_. We first investigated the impact of αCTD_003_ on the cytotoxicity of both TcdB_003_ and TcdB_027_ and found that treatment with αCTD_003_ neutralized the cytotoxic and cytopathic effects of TcdB_003_ (Fig. 4A). However, αCTD_003_ caused no detectible reduction in the cytotoxicity of TcdB_027_ (Fig. 4A). ELISA analysis confirmed that while αCTD_003_ was only able to neutralize TcdB_003_ in cell culture, the polyclonal serum could recognize both TcdB_003_ and TcdB_027_ in vitro (Fig. 4B). When the αCTD_027_ antibody was used in the neutralization assay, we found no protection against either TcdB_003_ or TcdB_027_, although the serum strongly reacted with both forms of the toxin as determined by ELISA (Fig. 4A and 4B).

The data shown in Fig. 4 suggested that CTD_027_ and CTD_003_ differ in their profile of neutralizing epitopes (i.e. sequences where antibody binding blocks intoxication). It was also possible that TcdB_027_ shared the same sequences of TcdB_003_ neutralizing epitopes, but, unlike TcdB_003_, TcdB_027_ did not depend on these regions for cellular intoxication. To address this alternative explanation, serum against CTD_003_ was incubated with a 100-fold excess of CTD_003_ or CTD_027_, and the mixture was tested for its ability to neutralize cytotoxicity of TcdB_003_. We reasoned that if CTD_027_ contains sequences that are targets for antibody-mediated neutralization of TcdB_003_ then the preincubation with CTD_027_ should prevent the antiserum from neutralizing TcdB_003_. As expected, the addition of CTD_003_ in the neutralization assay resulted in the inhibition of antibody activity and a return to full cytotoxicity of TcdB_003_ (Fig. 4C and 4D). In line with the possibility that TcdB_027_ contains sequences that are neutralizing epitopes in TcdB_003_, preincubation with CTD_027_ also blocked the neutralizing effects antiserum against TcdB_003_ (Fig. 4C and 4D).

### Fine Specificity Mapping of Antibody Responses Reveals Unique Epitope Differences between TcdB_003_ and TcdB_027_

**Figure 5:**
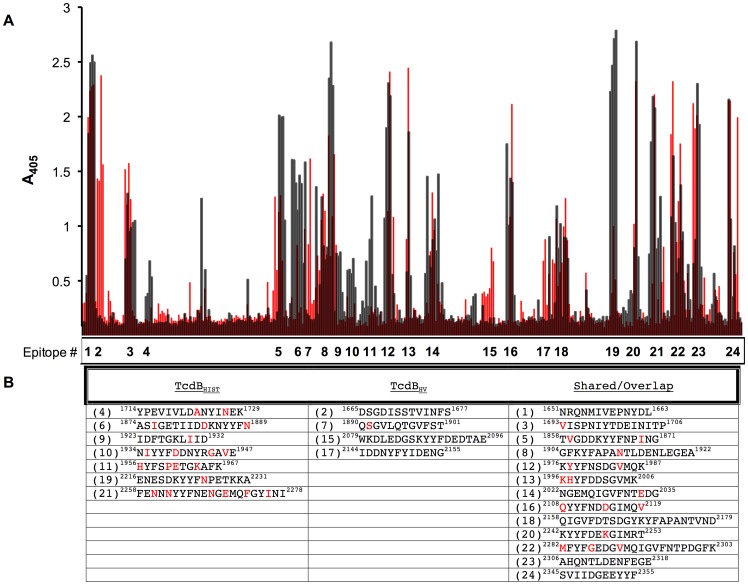
Identification of unique and shared epitopes between TcdB_003_ and TcdB_027_ using synthetic peptide ELISAs. Solid phase epitope mapping of αCTD_003_ (black) and αCTD_027_ (red) rabbit sera binding to overlapping decapeptides of the TcdB_003_ CTD. (a) Peptides from the CTD_003_ were constructed spanning amino acid 1651 through 2366, and the bars indicate the magnitude of reactivity of the sera to overlapping peptide sequences from the CTD of TcdB_003_. Reactivity is shown for αCTD_003_ (black) and αCTD_027_ (red), and represents an average of sera from 2 rabbits per group. (B) The peaks were numbered and identified as either unique to αCTD_003_ (left), unique to αCTD_027_ (middle), or overlapping/shared between αCTD_003_ and αCTD_027_ (right). The amino acid location of each epitope is indicated, as well as the sequence of the peptides in TcdB_003_, with amino acids that vary in TcdB_027_ identified in red.

The data from the analysis of antiserum against the two forms of TcdB suggested there is likely to be shared epitopes between the two proteins, but the extent of shared and unique epitopes was difficult to predict. In order to begin to identify shared and unique epitopes between TcdB_027_ and TcdB_003_ we used solid phase peptide based ELISAs to map antibody reactive sequences in the CTD of TcdB. In all, 358 decamer peptides, overlapping by 8 residues and covering the entire CTD_003_ sequence, were synthesized and tested for reactivity to CTD_003_ and CTD_027_ sera. Sera was collected from rabbits immunized with CTD_003_ or CTD_027_ (n = 2), and when we compared the peptides recognized by αCTD_003_ to those recognized by αCTD_027_ we found an overall difference in the pattern of peptides recognized by antisera from the 2 groups (Fig. 5). Each serum sample was analyzed individually, and the average response of αCTD_003_ and αCTD_027_ to the CTD_003_ peptides is shown in Fig. 5. The analysis identified identical epitopes, overlapping epitopes, and epitopes unique to each form of the toxin. The analysis identified approximately 7 regions that were recognized only by αCTD_003_ (Fig. 5). The analysis also found 4 regions recognized by only αCTD_027_ and 13 regions where there was overlap or exact matches in the epitopes recognized by both sera (Fig. 5). The majority of the peptides identified are localized in the CROP domain, and many of the epitopes that differ in recognition between αCTD_003_ and αCTD_027_ are located sequentially, within the first seven repeats of the CTD. As summarized in Fig. 5, peptides recognized by only the αCTD_003_ serum were variable regions between the two toxins, with as many as 6 amino acid differences as in the case of peptide 21. In contrast, the peptides recognized by only αCTD_027_ were highly conserved between the two forms of TcdB, with only one peptide (#7), with a single amino acid change. These data suggest that sequence variation of TcdB_027_ impacts antibody recognition of sequential epitopes and may contribute to differences in conformational epitopes as well.

### Mouse Antiserum against ToxoidB_027_ Is Cross-Protective In Vitro and In Vivo

**Figure 6:**
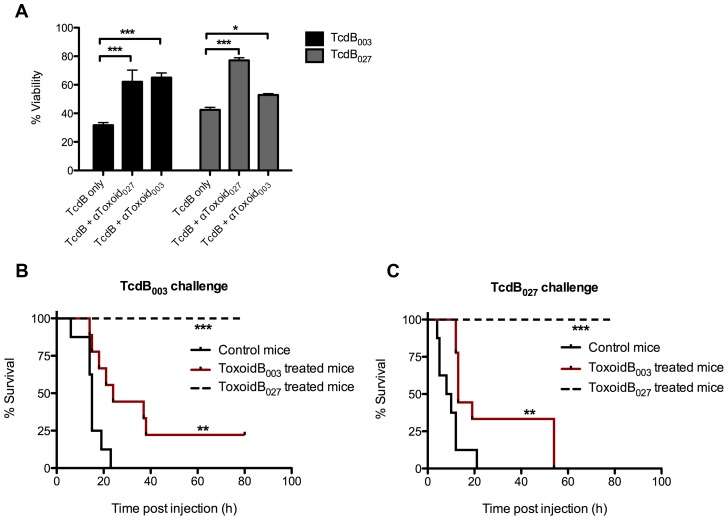
Protection against TcdB in vivo and in vitro after immunization with ToxoidB_027_. (A) Percent viability of CHO cells treated for 24 hrs with TcdB_003_ (black) or TcdB_027_ (gray) alone or after preincubation for 30 minutes with αToxoidB_003_ antiserum or αToxoidB_027_ antiserum. Cell viability was determined by WST-8 staining and the error bars represent the standard deviation from the mean of three samples. ***p<0.001, *p<0.05 (B–C) Kaplan-Meier graphs showing the time to death of C57Bl/6 mice that were injected intravenously with a 2×LD_100_ of TcdB_003_ (A) or TcdB_027_ (B) after immunization with ToxoidB_003_ (red), ToxoidB_027_ (dashed), or control peptide (black) (n = 9). Log-rank analysis performed using GraphPad Prism, *** p<0.001, ** p<0.01.

The observation that the CTD of TcdB_027_ is a poor target for the production of antibodies that prevent toxicity on CHO cells, raised concerns about the overall antigenicity of TcdB_027_. The majority of the amino acid sequence variation between TcdB_003_ and TcdB_027_ occurs in the CTD, so we reasoned that producing antibodies using the holotoxin as an antigen could have better potential to be broadly neutralizing. Both TcdB_003_ and TcdB_027_ were inactivated using formaldehyde to create ToxoidB_003_ and ToxoidB_027_. These toxoids were used as antigen to immunize mice and test for protective antibodies against TcdB. After two subsequent boosts, serum was collected from the mice, and the neutralizing effects were tested in vitro. The data in Fig. 6A shows that the mouse antiserum toward ToxoidB_027_ protected against the cytotoxic effects of both TcdB_003_ and TcdB_027_, while anti-Toxoid_003_ was not cross-neutralizing and only maintained the cell viability of the CHO cells treated with TcdB_003_. The immunized mice were next tested for protection from TcdB in vivo, using a 2-fold minimum lethal dose of TcdB_003_ or TcdB_027_. Consistent with the in vitro neutralization data, all mice immunized with ToxoidB_027_ were completely protected from i.v. challenge of both TcdB_003_ and TcdB_027_ (Fig. 6B and 6C). Immunization with ToxoidB_003_ provided only a slight, yet significant protective effect, increasing the median survival from 15 h to 24 h in mice injected with TcdB_003_, but only from 9 h to 13 h in mice challenged with TcdB_027_ (Fig. 6B and 6C). Eventually, all of the ToxoidB_003_ mice succumbed to the effects of TcdB_027_, and only two ToxoidB_003_ mice were fully protected from TcdB_003_ (Fig. 6B and 6C). Whereas the antisera to the CTD of TcdB_027_ showed no effect, antibodies to the toxoid form of TcdB_027_ successfully inhibited toxicity, suggesting that the protective effect against TcdB_027_ is better conferred by the full-length toxin rather than the CTD in this system.

## Discussion

*C. difficile* infection is a complex illness commonly involving colitis and, in more severe cases, systemic complications [54], [55], [56]. In the current study we sought to determine how systemic complications vary between two forms of TcdB. To focus on the systemic events mediated by the different forms of TcdB, we bypassed the intestinal stage of this illness by directly administering toxin intravenously. This analysis found that TcdB_027_ was more lethal and caused more pronounced systemic damage than TcdB_003_. Further studies revealed this effect correlated with differences in the extent of specific cellular tropisms between the variants of TcdB. Assessing the CTD of TcdB found that this region may contribute to not only differences in tropism, but also accounts for a variability in the antigenic make-up of this domain. Collectively, the data support the notion that TcdB_027_ is not only more potent than TcdB_003_, but may have sequence alterations that prevent cross neutralization.

Several recent observations led us to predict that the increased virulence of *C. difficile* BI/NAP1/027 is due to altered TcdB activity. First, the sequence of TcdB, but not TcdA, varies between the two strains [35], [57]. Second, in cell culture systems, TcdB_027_ is more potent on a broad range of cell types [35], [47], [57]. Thus, we hypothesized that TcdB_027_ could have a lower lethal dose and cause more extensive tissue damage in vivo. Our findings support this hypothesis. When experiments compared the lethal doses of TcdB_027_ and TcdB_003_ the BI/NAP1/027 toxin was found to be 4 times more lethal than the ribotype 003 toxin (Fig. 1). More importantly, TcdB_027_-treated mice died much more quickly and, in some cases, in less than half the time than TcdB_003_-treated mice. In regards to the pathologies, TcdB_027_ clearly caused brain damage that was less prominent in mice treated with TcdB_003_ (Fig. 2). These findings provide insight into the differences in the in vivo effects of TcdB_027_ and TcdB_003_, and this variation in toxicity could contribute to more severe disease caused by recently emerged strains of *C. difficile*.

Very little is known about the underlying mechanisms of *C. difficile*-induced systemic damage and complications. The extent to which the pathologies observed in toxin-treated mice reflect systemic complications in humans is not known and there is clearly a need for more studies in this area. However, several reports make it reasonable to suspect the toxins contribute to the systemic complications in this disease [54], [55], [56]. The idea that toxin enters the bloodstream during disease is supported by recent work using a piglet model of *C. difficile* infection where TcdA and TcdB were detected in the bloodstream of the infected animals [52]. Other work has demonstrated that serum IgG, and not mucosal IgA, against the toxins correspond with protection against illness and relapse [58], [59], [60] further supporting the notion of systemic effects of these toxins. Thus, the more extensive systemic damage caused by TcdB_027_ may explain in part why *C. difficile* NAP1/BI/027 is associated with more severe disease.

Our previous studies found that TcdB_003_ is cardiotoxic and targets cardiomyocytes with an equal efficiency to TcdB_027_ [35], [61]. In vivo and in vitro data support the notion that the two forms are TcdB are very similar in their cardiotoxic effects, but the sequence differences in TcdB_027_ allow the toxin to target other tissues an cell types more effectively than TcdB_003._ Consistent with this idea, the TCD_50_ for TcdB_027_ and TcdB_003_ was found to be very similar on aortic endothelial cells, but substantially lower for TcdB_027_ on brain microvascular endothelial cells. Thus, the evidence to date supports a model where both forms of TcdB are cardiotoxic, but TcdB_027_ is more potent on other tissue and cell types.

The fact that TcdB_027_ is a more potent toxin than TcdB_003_ is now well established by several in vivo and in vitro analyses [35], [47], including the ones used in this study. Yet, the sequence changes accounting for these differences in activity have not been defined. There are 198 residue differences between TcdB_027_ and TcdB_003_ and each of the residues known to be critical for TcdB activities are conserved between the two forms of this toxin. In previous work we found that TcdB_027_ undergoes more complete autocleavage because it is able to engage intramolecular substrate more effectively than TcdB_003_ [36]. This implies the conformation of TcdB_027_ may be different than that of TcdB_003_. We have also shown that TcdB_027_ undergoes dramatic pH-dependent conformational changes more extensively and at a higher pH than TcdB_003_ [35]. Again, this is unlikely to be related to a single residue change and could be the result of the collective sequence differences.

The finding that antibodies against the CTD neutralized TcdB_003_ but not TcdB_027_ on CHO cells could be the result of TcdB_027_ using an alternative means of cell recognition. Interestingly, Olling et al. have reported that the CROP domain of TcdA is involved in cellular uptake of the toxin, but it is not entirely responsible for cell recognition and binding [49]. In a like manner, it is plausible that the role of the CTD has become less significant in TcdB_027_ and variations have little effect on the toxin. If so, TcdB_027_ could bind cells by an alternative manner, which helps explain the current data that TcdB_027_ has a broad effect in mice, as well as previous data that shows extensive necrosis in a zebrafish model of intoxication.

The data from the peptide arrays showed αCTD_003_ reactivity with many epitopes in which the sequence varied in TcdB_027_. Whether these sequence variations evolved as a way of allowing TcdB_027_ to avoid immune recognition or if this is a means of TcdB_027_ altering its activity, is not yet clear. If the former is true, it could be possible that a change to one single epitope could be responsible for the lack of neutralization of TcdB_027_. However, work by Torres and Monath suggests that while the CTD is quite antigenic, antibodies to a single peptide epitope fail to prevent cytotoxicity of TcdB [50]. Finally, in further support of the idea that the two toxins are not identical in their overall structure, three of the epitopes recognized by serum against TcdB_027_ were not recognized by serum against TcdB_003_ despite the fact that these sequences were the same (Fig. 5). The conformational differences in the two forms of TcdB could determine whether identical sequences are antigenic.

It is also important to consider this variation in the context of virulence of *C. difficile*, as well as vaccination. Our previous work suggests that TcdB_027_ enters cells more rapidly and efficiently than TcdB_003_ [35]. Given that the CTD is believed to facilitate interactions with the cell surface, it is possible that antigen recognition occurs, but the toxin overcomes this by utilizing a more effective mechanism of cell entry. Arguing against this possibility is the fact that we did not detect even a minor change in the rates of TcdB_027_-induced cell rounding or the overall level of cell killing. It's also important to note that our experiments involved preincubating TcdB_027_ with the antiserum. Therefore, if the toxin overcame the neutralizing effect by more efficient cell entry, we would expect to see at least a nominal change in toxicity, but this doesn't appear to be the case. We believe the reasonable explanation is that the neutralizing epitopes of TcdB_027_ are sufficiently altered to avoid toxin neutralization or that the toxin has a different mechanism of interacting with and entering the cell. These data also suggest successful vaccines targeting TcdB will need to include antigens from multiple forms of this toxin or, alternatively, be designed to target highly conserved neutralizing epitopes shared among variants of TcdB.

Although further studies are needed, the toxoid of TcdB_027_ could provide a vaccine that generates a broadly neutralizing response. Given that the CTD_027_ did not generate an antibody response that protected CHO cells from TcdB_027_, and past studies have found that TcdB toxoid is not a highly effective vaccine [62], [63], we were surprised to find the toxoid of TcdB_027_ stimulated a potent neutralizing response in mice. It has been known for many years that anti-serum does not cross neutralize TcdA and TcdB, making it reasonable to consider the possibility that anti-serum to the variant forms of TcdB also do not cross neutralize. This does not appear to be the case. As shown in Fig. 6, mice vaccinated with the toxoid form of TcdB_027_ were completely protected against both TcdB_003_ and TcdB_027_. In line with a prior study by Wang et al. [64], the toxoid of TcdB_003_ evoked only marginal immunoprotection against TcdB, and we found this to be true for mice challenged with either the historical or ribotype 027 form of the toxin. This raises the possibility that converting TcdB_003_ into a toxoid alters the protein in a way that reduces immunogenicity, but sequence differences in TcdB_027_ make this form of the toxin more effective as a toxoid.

Overall, these findings demonstrate critical differences between TcdB produced by ribotype 003 and ribotype 027 strains of *C. difficile*. The sequence variations in TcdB_027_ impact the toxin's cytotoxicity, lethality, and antigenic make-up, and likely contribute to the overall heightened virulence of *C. difficile* BI/NAP1/027 strains.

## Materials and Methods

### Ethics Statement

The animal immunization and toxin challenge studies were performed in strict accordance with the recommendations in the Guide for the Care and Use of Laboratory Animals of the National Institutes of Health. All animal procedures reported herein were approved by the Institutional Animal Care and Use Committee and Institutional Biosafety Committee at OUHSC (IACUC protocol # 09-092-I and 11-016-I). The procedures precluded the use of anesthesia for in vivo lethal challenge assays. To minimize pain and distress, the mice were monitored at least twice daily and any animals with signs of distress such as labored breathing, lethargy, inability to eat or drink, ruffled fur, disorientation, or loss of 20% body weight were euthanized immediately. This method was approved by the IACUC and monitored by a qualified veterinarian.

### Animals, Bacterial Strains, and Cell Culture

*C. difficile* VPI 10463, a ribotype 003 strain (produces TcdB with identical sequence to the 630/ribotype 012 strain), and *C. difficile* BI17 6493, a ribotype 027 strain (a gift from Dr. Dale Gerding), were used as sources of to purify TcdB_003_ and TcdB_027_ respectively.

Female BALB/cJ and C57B/6J mice (Jackson Laboratories), aged 8 weeks, were purchased from The Jackson Laboratories (Bar Harbor, ME) and handled in accordance with IACUC guidelines at University of Oklahoma Health Science Center.

Rat Brain Microvascular Endothelial Cells (RBMVEC) and Rat Aortic Endothelial Cells were a generous gift from the laboratory of Dr. Eric Howard (University of Oklahoma Health Sciences Center) and have been described previously [65], [66]. CHO-K1 cells were purchased from American Type Culture Collection (ATCC). RBMVEC and RAEC were grown in DMEM containing 10% FBS while CHO cells were grown in F12-K with 10% FBS. All cell types were used between passage 15–30, and were maintained in tissue culture treated T-75 flasks (Corning) at 37°C in the presence of 6% CO_2_.

### Production of Native Toxin, Toxoid Preparation, and Purification of Recombinant Tcdb Fragments

*C. difficile* was cultured using the dialysis method as previously described [35] and TcdB was isolated using anion-exchange (Q-Sepharose) chromatography in 20 mM Tris-HCl, 20 mM CaCl_2,_ pH 8.0, following a thyroglobulin affinity chromatography protocol to first remove TcdA [67]. Purification of TcdB was confirmed by visualization of a single 270 kDa band by SDS-PAGE, and LC/MS/MS analysis (University of Oklahoma Health Science Center).

Toxoid versions of TcdB_003_ and TcdB_027_ were prepared by mixing 500 µl of TcdB (0.4 µg/µl) into 500 µl of 8% formaldehyde with 8.5 mg of lysine to help prevent precipitation and aggregation of the formalinized protein [68], [69], and incubating at 37°C overnight. The volume was then brought up to 10 ml with PBS, yielding 20 µg/ml of ToxoidB in 0.4% formaldehyde with 0.425 mg/ml lysine. Both toxoid preparations lacked toxic activity as confirmed by the absence of cytopathic effects on CHO cells.

The CTD-encoding region of *tcdb* gene (YP_001087135.1: nucleotides 4961–7111) from the strain VPI 10463 was codon optimized and cloned into pET15b (Genscript). The CTD of the *tcdb* gene (YP_003217086.1: nucleotides 4961–7111) from the NAP1 strain was cloned from a pET15b plasmid containing full-length *tcd*b that had been codon optimized by Genscript. The CTD gene was amplified using primers 5′-GATCATATGCTGTATGTGGGTAACCG-3′ and 5′-AACGGATCCTTATTCGCTAATAACCA-3′ containing *Bam*HI and *Nde*1 sites for cloning into pET15b. The CTDs were expressed using *Escherichia coli* BL21 star DE3 (Invitrogen) at 16°C overnight and then purified by Ni^2+^ affinity chromatography (HisTrap, GE Life Sciences) resulting in proteins representing TcdB_1651–2366_ from both TcdB_003_ and TcdB_027_.

### Lethal Dose Determination and Organ Pathologies

To determine the differences in the minimum lethal dose of TcdB_003_ and TcdB_027_, 100 µl of TcdB_003_ or TcdB_027_ dilutions in phosphate-buffered saline was injected intravenously into the tails of BALB/cJ mice using a 27-gauge needle. Twenty mice were given TcdB_003_ in groups of 4, receiving doses of 2 µg, 1 µg, 500 ng, 100 ng, and 50 ng. Twenty additional mice were injected with doses of 200 ng, 100 ng, 50 ng, 25 ng, and 12.5 ng of TcdB_027_ (n = 4). The animals were monitored for up to 7 days post challenge for toxin effects and mortality, and mice were euthanized if they became significantly distressed or moribund. Survival was graphed using Kaplan-Meier analyses on GraphPad Prism (GraphPad Software, Inc., La Jolla, CA).

Immediately after death, the mice were dissected and major organs and tissues were submerged in formalin fixative overnight. Tissue sectioning, slide preparation, H&E staining, and pathology analysis was performed by the Department of Comparative Medicine at OUHSC.

### Animal Immunizations and TcdB Challenges

Two rabbits per group were immunized with 0.1 mg of the CTD fragment of TcdB_003_ or TcdB_027_ in complete Freund's adjuvant on day 1 and boosted with 0.1 mg in incomplete Freund's adjuvant on days 14, 21, and 49. Blood samples were collected on days 0, 35, and 56. These experiments were carried out by Cocalico Biologicals Inc. (Reamstown, PA).

BALB/cJ mice (20 mice each for ToxoidB_003_ and ToxoidB_027_) were injected in equal portions subcutaneously and intraperitoneally with 2 µg of toxoid in PBS emulsified 1∶1 in 100 µl of complete Freund's adjuvant on day 1 and boosted with 2 µg in incomplete Freund's adjuvant on day 10. Control mice were similarly immunized and boosted using an unrelated peptide. Blood samples were collected via tail bleeds on day 0 and 24, and each bleed was tested by ELISA to evaluate toxoid response.

After completion of the immunizations, the mice were subjected to i.v. challenges of TcdB_003_ and TcdB_027_. Each immunization group (ToxoidB_003_, ToxoidB_027_, control) contained 20 mice, and 9 from each group were injected via the tail vein with a 2-fold lethal dose of either TcdB_003_ or TcdB_027_. The previously established minimum lethal dose was used to set the 2×LD_100_ at 200 ng per mouse for TcdB_003_ and 50 ng per mouse for TcdB_027_. The remaining 2 mice from each group were euthanized and exsanguinated for serum collection. The animals were monitored for up to 7 days post challenge for toxic effects and mortality, and mice were euthanized if they became significantly distressed or moribund. Survival was graphed using Kaplan-Meier analyses and compared with the Log-rank test on GraphPad Prism (GraphPad Software, Inc., La Jolla, CA).

### Characterization of Antibody Responses

Direct antigen ELISAs were used to measure the antibody reactivity in animal sera. 1 µg of purified TcdB or CTD fragment was coated per well in polystyrene plates at 4°C overnight. The plates were washed and blocked with 0.1% BSA in PBS for 1 h at room temperature. Then, the rabbit sera diluted at 1∶100 and 1∶1000 in PBS-Tween with 0.1% BSA was added in triplicate and incubated for 2–3 h at room temperature. Plates were washed with PBS-Tween and incubated with anti-rabbit IgG conjugated to alkaline phosphatase (Jackson ImmunoResearch Laboratories, Inc) at a dilution of 1∶5,000 for 3 hours at room temperature then washed and developed with p-Nitrophenyl Phosphate substrate (Sigma). Plates were read at 405 nm using a Tecan-infinite plate reader (Tecan Group, Ltd.). Plates were read when the positive control reached an OD of 1.0 and the assay was considered invalid if the negative control was over OD 0.2.

### Cytotoxicity and TcdB Neutralization Assays

Cells were seeded in 96 well plates at a density of 1–2×10^4^ cells per well in DMEM or F12-K (ATCC) containing 10% FBS (ATCC). For TcdB sensitivity measurements on endothelial cells, dilutions of TcdB_003_ or TcdB_027_ were added to each well in triplicate, and the cells were incubated 24 h and cell viability was measured by CCK-8 (Sigma). In order to measure neutralization of TcdB, a 1∶10 dilution of serum raised in rabbits against the CTD or mouse serum to the toxoid was preincubated with 37 pM TcdB_003_ or TcdB_027_ alone, or with 3.7 nM CTD_003_ or CTD_027_, for 1 h at 37°C in F12-K media (ATCC). CHO cells were treated with the toxin/antiserum mixture or toxin alone and incubated at 37°C for up to 24 h. Cells were analyzed under the microscope for cell rounding at 2–4 h and cell viability was measured at 24 h using a CCK-8 assay according to manufacturers instructions (Sigma).

### Fine Specificity Epitope Mapping with Solid-Phase Peptide ELISAs

The 358 decapeptides overlapping by 8 amino acids covering the length of the CTD region from TcdB_003_, were covalently synthesized on polyethylene solid phase supports (pins) as previously described and used to assay antibody specificity with a modified ELISA assay [70]. Blocking was performed in 3% milk in PBS for 1 h at room temperature, then the peptides were incubated in 100 µl/well of sera diluted 1∶100 in 3% milk-PBS with 0.05% Tween for 2 h at room temperature. The pins were washed 4 times for 8 min with mild agitation in PBS-Tween and then incubated with 100 µl/well of a 1∶5,000 dilution of anti-rabbit IgG conjugated to alkaline phosphatase in 3% milk-PBS with 0.05% Tween at 4°C overnight (Jackson ImmunoResearch Laboratories). Next, washes were performed as previous and the peptide ELISAs was developed using 100 µl/well of a 1 mg/ml solution of p-nitrophenyl phosphate dissolved in 150 mM carbonate buffer pH 10.4 containing 100 mM glycine, 1 mM MgCl_2_ and 1 mM ZnCl_2_. The absorbance was read at 405 nm using a Tecan-infinite plate reader (Tecan Group, Ltd.), and the results were normalized to the standard positive control peptide having an OD of 1.0. Positive epitopes were defined as at least two consecutive peptides with an OD greater than 2 standard deviations above the mean of pre-bleed serum.

### Accession Numbers

Relevant SwissProt accession numbers are P18177 (TcdB_003_/CTD_003_), P16154 (TcdA_003_), C9YJ35 (TcdB_027_/CTD_027_), C9YJ37 (TcdA_027_),
